# Contact-Dependent Growth Inhibition in Bacteria: Do Not Get Too Close!

**DOI:** 10.3390/ijms21217990

**Published:** 2020-10-27

**Authors:** Larisa N. Ikryannikova, Leonid K. Kurbatov, Neonila V. Gorokhovets, Andrey A. Zamyatnin

**Affiliations:** 1 Institute of Molecular Medicine, Sechenov First Moscow State Medical University, Trubetskaya 8/2, 119991 Moscow, Russia; gorokhovets@gmail.com; 2 Orekhovich Research Institute of Biomedical Chemistry, Pogodinskaya 10/8, 119991 Moscow, Russia; kurbatovl@mail.ru; 3 Belozersky Research Institute of Physico-Chemical Biology, Lomonosov Moscow State University, Leninskie Gory 1/40, 119992 Moscow, Russia; 4 Department of Biotechnology, Sirius University of Science and Technology, 1 Olympic Ave, 354340 Sochi, Russia

**Keywords:** contact-dependent growth inhibition (CDI), bacterial communities, intra-and interspecies competition, antibacterial toxins

## Abstract

Over millions of years of evolution, bacteria have developed complex strategies for intra-and interspecies interactions and competition for ecological niches and resources. Contact-dependent growth inhibition systems (CDI) are designed to realize a direct physical contact of one bacterial cell with other cells in proximity via receptor-mediated toxin delivery. These systems are found in many microorganisms including clinically important human pathogens. The main purpose of these systems is to provide competitive advantages for the growth of the population. In addition, non-competitive roles for CDI toxin delivery systems including interbacterial signal transduction and mediators of bacterial collaboration have been suggested. In this review, our goal was to systematize the recent findings on the structure, mechanisms, and purpose of CDI systems in bacterial populations and discuss the potential biological and evolutionary impact of CDI-mediated interbacterial competition and/or cooperation.

## 1. Introduction

**Figure 1:**
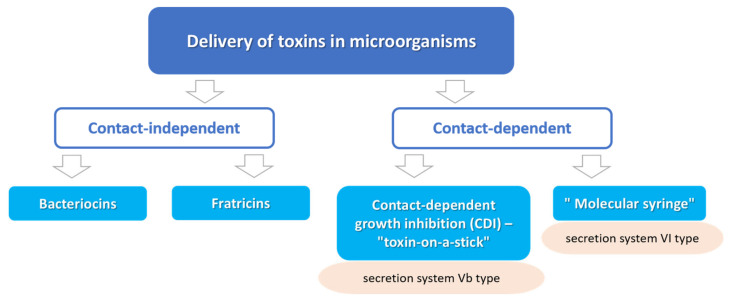
Delivery of toxin effectors into target cells in microorganisms.

Like most living organisms, the inhabitants of the microworld (including bacteria) prefer to form mono-species or more complex multi-species communities. In these communities, bacteria of one species can collaborate against other species, distribute responsibilities, and even sacrifice a part of the population for the survival of the species due to changing environmental conditions [1,2,3,4,5]. One of the ways a bacterial cell can adapt to its environment is the production of effector molecules (“toxins”), some of which are designed to interact with the host cells, while others are aimed at regulating relationships with other inhabitants of the microbiome. These effector molecules, usually proteins, are exported from the cell to the environment, where they diffuse freely until meeting a target cell that is both sensitive to them and has a suitable receptor on its surface. By binding to the receptor, toxins interact with the cell wall or other cell structures leading to their damage and injury or even death of the target cell. This is a mode of action of bacteriocins (bacterial antimicrobial peptides, AMP) [6,7] or fratricins (AMP or proteins produced by isogenic cells of the same species) [8,9,10]. In some cases, effector toxins can be delivered directly to the neighboring bacteria cells as a result of direct physical contact with them. As such, in the following, we discuss contact-dependent interactions [11,12,13,14,15,16] (Figure 1).

Contact-dependent competitive interaction is mediated by two specialized secretion systems: type V (T5SS) or VI (T6SS). The type VI secretion system (referred also to as the “molecular syringe” or “molecular crossbow”) is a multi-protein complex in the cell’s periplasm. The key component of this complex is a needle-like structure similar to the contractile tail of the bacteriophage T4. When the outer sheath, which plays the role of a kind of piston, contracts, the “needle” is pushed out, puncturing the membrane of a neighboring cell and releasing toxic effectors into its cytoplasm. This mechanism is found in about a quarter of all bacterial species and is described in detail in several excellent reviews [17,18,19,20]. In our review, we consider the phenomenon of contact-dependent growth inhibition (CDI) mediated by the Vb type secretion system. Despite the general similarity in purpose (delivery of a toxin to a neighboring cell) and design (a multi-protein “docking” complex for making contact with a target cell) with a “molecular syringe”, a notable feature of the CDI system is the need for specific receptors on the surface of the target cell to interact with the receptor recognizing site of the CDI toxin [21,22,23,24,25,26,27,28].

Since its discovery in 2005, CDI has been detected in many Gram-negative bacteria, including clinically significant human pathogens, such as *Acinetobacter baumannii*, *Pseudomonas aeruginosa*, or *Neisseria meningitidis* [23,25,29,30,31]. In recent years, many new works investigating this phenomenon have appeared, which is explained by the drive to find new approaches to the manipulation of microbial communities to address growing number of antibiotic resistant bacteria. In this review, we provide an overview of what is currently known about the molecular mechanism and the biological role of CDI mediated competition and cooperation in bacterial species.

## 2. Contact-Dependent Growth Inhibition in *Escherichia coli*

The phenomenon of contact-dependent growth inhibition was first discussed in 2005 by D. Law’s group, in the experiments on suppressing the growth of the *E. coli* K-12 indicator strain by *E. coli* EC93 isolate [32,33]. *E. coli* EC93 prevailed in the feces of a family of laboratory rats, and this fact gave rise to the assumption that this strain has a competitive advantage over other intestinal inhabitants. As is well known, *E. coli* strains can produce soluble antimicrobial peptides (colicins), but the inhibitory activity of *E. coli* EC93 was not associated with AMP production. The growth of the indicator strain was not inhibited in the EC93 growth medium in the absence of living cells. Inhibitory activity did not appear even in the presence of mitomycin, an inducer of colicin synthesis. Separation of the test EC93 and indicator K-12 strains by a 0.4 micron porous plastic membrane permeable to the components of the culture medium (but not to bacterial cells) showed that in the absence of direct physical contact, K-12 growth inhibition, was not observed; however, using a membrane with a large (8 mm) pore diameter resulted in a significant (1000 times) suppression of the growth of the indicator strain [32].

**Figure 2:**
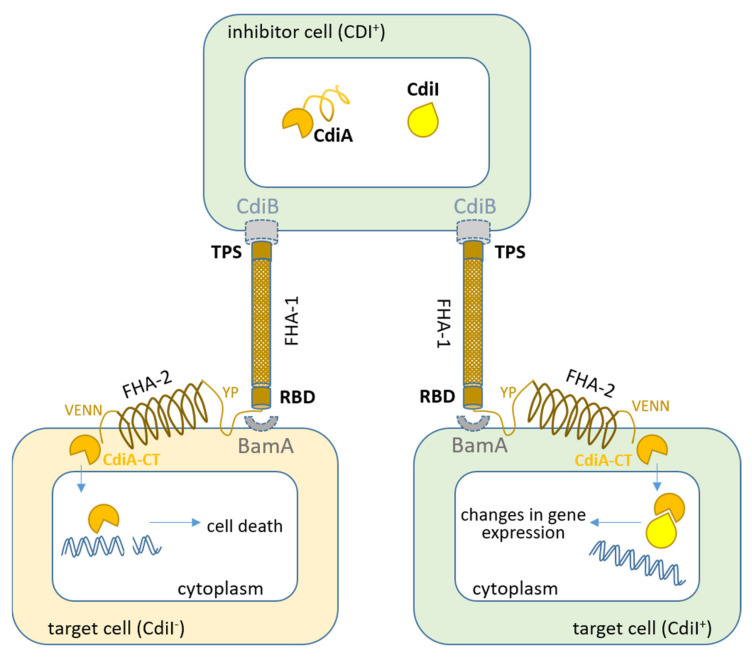
The contact-dependent growth inhibition (CDI) model of toxin delivery into a neighboring cell. The C-terminal effector domain (CdiA-CT) (toxin) is delivered to the target cell via specific membrane receptors (left part of the figure). In the cytoplasm, CdiA-CT degrades the nucleic acids (DNA or RNA) of the target cell leading to growth arrest and cell death. When entering a cell that produces CDI proteins (right part of the picture), CdiA-CT is inactivated by binding to the corresponding immune proteins CdiI. It was assumed that the complex of CdiA-CT/CdiI with nucleic acids and/or target cell proteins induces changes in gene expression leading to phenotypic changes [13,35].

The CDI^+^ phenotype was established to be mediated by the *cdiBAI* gene cluster [32]. CdiA adhesin is a ~320 kDa protein, consisting of three domains: a large conserved N-terminal domain (NtD) with a triple-stranded beta-helix structure, a receptor-binding domain (RBD), and a smaller C-terminal effector domain (CdiA-CT) [25,34,35]. CdiA is secreted through CdiB, a β-barrel outer membrane protein of the Omp85-TpsB superfamily. It is assumed that CdiA can extend from the surface of a CDI^+^ cell for some (about 30 nm) distance to bind its receptor-binding site to a specific receptor of the target cell [35]. Upon contact with the target, CdiA autoproteolytically cleaves its effector part, CdiA-CT, which translocates into the competitor cell [32,36] leading to disruption of intracellular processes (Figure 2). Due to its peculiar structure, CdiA was named “toxin-on-a-stick” [33].

Immunity to its own toxin is provided by CdiI, a small (8.5 kDa) protein located on the inner membrane, where it can form a tight complex with CdiA-CT [32,37].

## 3. “Toxin-on-a-Stick”: The Structure of the CdiA Protein

**Figure 3:**
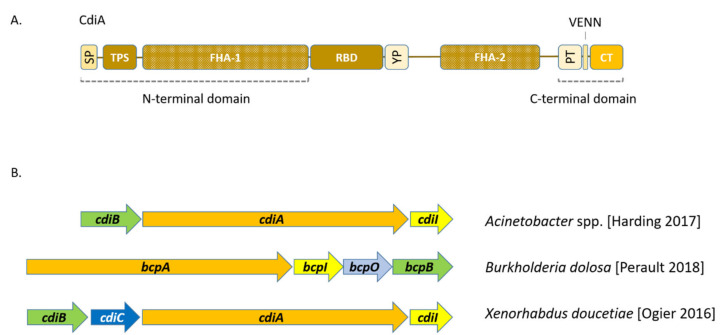
(**A**) CdiA domain architecture in *E. coli*. SP—signal peptide; TPS—two-partner secretory system; FHA-1,2—peptide repeats (regions 1, 2); RBD—receptor-binding domain; YP—conservative domain with a predominance of Tyr (Y) and Pro (P). PT—pre-toxin module. VENN is a conservative peptide motif that separates the pre-toxin module and the variable CdiA-CT regions. CT—toxin CdiA-CT. (**B**) Structure of the CDI locus in different species (see text).

CdiA is a key protein in the CDI system. These proteins differ significantly in size and sequence in different representatives of the bacterial domain: from ~180 kDa in the *Moraxella* species to more than 630 kDa in *Pseudomonas* [38]. Despite the significant heterogeneity, these proteins have a similar domain architecture. As mentioned above, CdiA consists of three parts: the N-terminal (NtD) and C-terminal (CdiA-CT) domains, as well as the receptor-binding domain (RBD) (Figure 3A). The N-terminal part carries a signal peptide for Sec-dependent translocation into the periplasm, and a conserved two-partner secretion (TPS) transport site for CdiB-mediated export across the outer membrane [35,39]. Most of the N-terminal domain consists of peptide repeats, in which, the filamentous hemagglutinin (FHA) FHA-1 motif dominates. Structural modeling confirmed by electron microscopy data showed that FHA-1 repeats form an elongated β-helix, in which each link consists of an average of 20 amino acid residues and adds 4.8 Å to the helix length [35]. The C-terminal part of this protein also contains some number of FHA repeats (FHA-2), which presumably differ from FHA-1.

The receptor-binding domain is located between FHA-1 and FHA-2. The amino acid sequence of this site may differ quite significantly even between closely related CdiA [34]. The BamA—a key subunit of the outer membrane β-barrel assembly machine (BAM) complex [40,41]—as well as heterotrimeric complexes of OmpC and OmpF porins [42] and nucleoside transporter Tsx [34], were identified as corresponding receptors for various RBDs. Next to the RBD site, there is a conserved YP region (185 AA residues) enriched for Tyr (Y) and Pro (P) residues relative to the surrounding FHA repeat domains. The role of the YP domain is not yet fully understood; this site probably plays the role of a holder stabilizing CdiA on the outer membrane [35].

The C-terminal domain of CdiA is highly polymorphic and contains various types of toxin modules. Thus, CdiA-CT^EC93^ from the *E. coli* EC93 strain acts as an ionophore toxin dissipating the proton motive force of the targeted cell [43], whereas CdiA-CT from the uropathogenic strain *E. coli* 536 (CdiA-CT^UPEC536^) and enterohemorrhagic *E. coli* 869 (CdiA-CT^EC869^) are nucleases that cleave tRNA inside the target cell [28,29,44]. To date, at least 18 variants of CdiA-CTs are known for *E. coli* [35]. Most of the CdiA-CT toxins studied demonstrate nuclease activity.

On the N-terminus, the CdiA-CT region is demarcated by the VENN conserved peptide motif, which is part of a larger PT (“pre-toxin”) domain in *E. coli*. The function of the PT domain has yet to be clarified, but its location suggests a role in the autoproteolytic release of CdiA-CT toxin [45].

Taking into account the variations in composition, *E. coli* CdiA proteins are usually divided into five or more classes. Classes I–III are defined by the amino acid sequence of the RBD domain [46], which corresponds to three different types of receptors on the surface of the target cell: BamA (class I), OmpC/OmpF (class II), or Tsx (class III) [34,40,41,42].

## 4. CDI Systems in Other Bacterial Species

Both functional CDI systems and genes encoding CDI proteins are identified in many bacterial species [29,45]. These genes are typically found in the “auxiliary” genomes, often within genomic pathogenicity islands or plasmids, therefore not all strains of a given species necessarily carry *cdi* genes [25,29,30]. Thus, for *E. coli*, CDI loci were found in about 16% of all genomes studied [25]. This type of species competition has been discovered in several Gram-negative microorganisms including *E. coli* [28,30,32,35,36,37,38,40,41,42,43,44,47,48,49,50,51], *Burkholderia* spp. (*Burkholderia thailandensis, Burkholderia dolosa*) [52,53,54,55,56,57,58,59], *N. meningitidis* [60], *P. aeruginosa* [61,62,63,64], and *Acinetobacter* spp. (*A. baumannii*, *Acinetobacter baylyi*) [65,66,67,68] (Table 1), while proteins similar to CdiA-CT toxin were identified also in Gram-positive species including *Bacillus*, *Listeria*, *Clostridium*, and *Streptococcus* [31]. Such proteins derived from *Bacillus subtilis* or *Bacillus cereus* and expressed in *E. coli* showed RNase activity and suppressed the growth of wild *E. coli* strains [31]. Additionally, proteins of the LXG family were described which are similar in structure to CDI toxins. These proteins exported into adjacent bacterial cells by the Esx pathway can mediate contact-dependent interspecies antagonism in Gram-positive bacteria [69].

The organization of the CDI locus of *Burkholderia* spp. slightly differs from that described for *E. coli*; therefore, the CDI systems are typically divided into the “*E. coli*” or “*Burkholderia*” type. *Burkholderia*-type CDI systems are encoded by the *bcpAIOB* gene cluster. As in the case of *E. coli*, the *bcpA* and *bcpB* encode the exoprotein and TPS transporter, respectively, whereas *bcpI* encodes the immunity protein (Figure 3B). The *bcpO* gene, located between *bcpI* and *bcpB*, encodes a small protein whose function is not still precisely established [58] (see below). As in *E. coli*, the amino acid sequence of the N-terminal domain (about 2800 amino acid residues) of BcpA toxin is conserved, whereas the C-terminal part (~300 amino acid residues) varies greatly in closely related species [27,29]. The variable BcpA-CT region in *Burkholderia*-type systems is demarcated by an NX(E/Q) LYN (VENN in *E. coli*) motif [52]. According to the amino acid sequences of the BcpB and BcpO proteins, as well as the conserved part of BcpA, the *Burkholderia*-type CDI systems are classified into two distinct phylogenetic groups (class I and class II). The *bcpO* genes across different *Burkholderia* class I alleles are almost identical and can presumably encode small lipoproteins localized on the outer membrane. The deletion of this gene leads to some decrease in the effectiveness of growth inhibition. The “*bcpO*” genes of class II are not similar across different alleles and have no similarity to class I *bcpO* genes; the deletion of these genes does not significantly affect the inhibition of growth [29,52,58].

CDI loci have been identified via bioinformatics approaches in more than 100 *P. aeruginosa* genomes [63]. A second CDI locus was found in 81% of the genomes. Based on a set of pre-toxin protein motifs demarcating the highly variable C-terminus of CdiA (like the VENN motif in *E. coli*), CdiA can be classified into five subtypes in *Pseudomonas* species: WVHN (class I), VENN (class II), LYVT (class III), DAMV (class IV), and NEALV (class V) [61,63]. Of these, *P. aeruginosa* CdiA proteins are restricted to the class II and class V groups. More than 40 variants of CDI systems have been identified within the genus *Acinetobacter* [67]. These variants form two distinct groups based on the structure of the CdiA protein. Type II CdiAs are giant proteins (3711–5733 AA residues) with long arrays of 20-mer repeats, whereas type I CdiAs are significantly smaller (1900–2400 AA residues) and lack repeats, but have central heterogeneity (HET) regions that vary in size and sequence and can probably be exchanged between CdiA proteins [67].

In *Xenorhabdus doucetiae*, a bacterial symbiont of entomopathogenic nematodes, the CDI locus belongs to the “*E. coli*” type, but additionally includes a gene encoding the CdiC protein (Figure 3B); however, its function is still unknown. This gene is co-transcribed with other CDI genes in all phases of microorganism growth, and its product could probably play a role in the activation of the CDI toxin or contribute to the interaction of CdiA or CdiB with the membrane [71].

In general, *cdiB*, *cdiA*, and *cdiI* typically constitute the minimal core of different CDI systems, though some CDI loci contain additional genes, which are probably important for their functionality (like *bcpO* in *Burkholderia* or *cdiC* in *X. doucetiae*). The encoding sequence of CdiA toxin is usually closely linked to the downstream immunity *cdiI* gene forming a toxin/immunity pair. CdiA-CT and CdiI sequences are extraordinarily polymorphic between bacteria; however, the related CdiA-CT/CdiI pairs can be often found in diverse bacterial clades. This suggests that CdiA-CT/CdiI pairs are horizontally exchanged between bacteria. This hypothesis is supported by work with experimentally generated CdiA chimeras; thus, the CdiA-CT^EC93^ toxin region can be fused to CdiA^UPEC536^ at the conserved VENN peptide motif to generate a functional CdiA protein [29]. This means that there is a large repository of toxins and corresponding immunity genes shared by a variety of toxin delivery systems.

Further evidence of horizontal exchange is the tandem arrays of “orphan” *cdiA-CT/cdiI* gene pairs often found downstream of *cdiBAI* gene clusters (see below).

## 5. Harpoon with Replaceable Tips: Orphan CDI Modules

**Figure 4:**
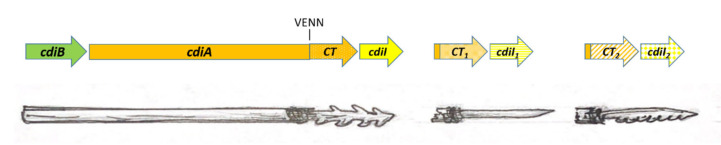
Orphan toxin/immunity modules. Orphan *cdiA-CT/cdiI* modules include conserved sequences upstream of the VENN-encoding region. Presumably, homologous recombination can occur with a full-size *cdiA* gene, so the orphan modules can be considered as a set of toxins “tips” for the protein harpoon.

In addition to the full-length *cdiBAI* operon, the genomes of many bacteria contain fragmentary sets of genes similar to those that encode CdiA-CT. These fragments usually lack the translation initiation sites, so it is unclear how these toxins should be synthesized. A part of the conserved *cdiA* coding sequence of varying lengths can be found upstream of the VENN-encoding region, but the sequences of the signal peptide or TPS transport domain are usually absent; therefore, the export of this toxin from the cell is hardly possible. *cdiA-CT* homologs are almost always associated with the adjacent genes of the corresponding *cdiI* immunity proteins (Figure 4). Such fragments were called “orphan” CDI modules [25,30]. Within orphan modules, there are usually sequences homologous to transposases and integrases, as well as elements of insertion sequences. This suggests that bacteria can probably replace its original *cdiA-CT/cdiI* module with a new one, by homologous recombination at the N-terminus [70]. Thus, orphan CDI modules can be considered as a kind of the stockpile of “replaceable” toxin modules in the bacteria’s arsenal.

When expressed in *E. coli*, many orphan toxins demonstrate the growth inhibition activity that can be blocked by the corresponding (located nearby) immunity protein [30]. Interestingly, orphan modules of some species can be discovered as part of the full-size CdiA protein of other species.

## 6. What Are CDI Systems Designed for?

One of the most interesting questions is what are CDI systems for?

Bacterial CDI systems were first described in the terms of their ability to mediate interbacterial competition [32]; however, the relevance of this competition is still not definitively clear, and there is increasing evidence that CDI toxins can play different roles in bacterial biology.

### 6.1. Interbacterial Antagonism

The key point in the definition of biological roles of the bacterial CDI systems is a question of the specificity of CDI toxin translocation into the recipient cell. Contact occurs by binding the RBD site of CdiA to specific receptors on the surface of the target cell. The diversity of a pool of bacteria-recipients is thus limited to those who produce appropriate receptor variants. The toxin delivery by class I CdiA proteins that bind to the outer-membrane BamA is strictly species-specific and limited to the *E. coli* population [41]. BamA is a conserved protein involved in outer membrane protein folding, and its homologs are universally found in Gram-negative bacteria [72,73,74]; however, extracellular loops 6 and 7 of this protein are highly variable within the species, hindering an interaction between receptor and RBD sites of CdiA of species other than *E. coli*. The most efficient receptor–toxin pairs are probably encoded by the same bacterial strain, which suggests that CDI systems can be preferentially aimed at their own strain or closely related bacteria. Indeed, closely related neighbors may appear to be more threatening competitors within the same ecological niche than more distantly related species.

Beck et al. demonstrated that the class II CdiA proteins that bind to OmpF/OmpC are strain-specific and able to discriminate between different strains of *E. coli*, with a preference for their “own” strain over others [42]. On the contrary, Virtanen et al. found, that the *E. coli* class II CdiA RBDs allow for the delivery of toxic effectors into many different *Enterobacteriaceae* spp., including *Enterobacter cloacae* and *Enterobacter aerogenes*, *Klebsiella pneumoniae*, and *Salmonella typhimurium*, suggesting that class II CDI is a broad-range inter-species competition system [46]. Similar findings were made in other works: thus, over-expression of the CdiBAI module in *E. cloacae* leads to an inhibition of *E. coli* growth [70], while CDI systems of *Burkholderia pseudomallei* allows for the delivery of effector protein to the closely related *B. thailandensis* [57].

To summarize, we can say that the participation in species antagonism is probably an important function of CDI systems, at those toxins produced by means of CDI are likely exchanged among “self” (isogenic or closely related) cells possessing the necessary receptors for toxin translocation rather than delivered to “non-self” competitors. However, sibling bacteria can produce the cognate immunity proteins that protect them from their own toxins; therefore, the hypothesis of a possible signaling function of CDI toxins arise [13,44].

### 6.2. To Kill or to Kiss? Contact-Dependent Signaling

As suggested above, CDI systems can not only antagonize competitors but also mediate communication and signaling exchange between bacteria that produce identical toxin/antidote pairs. Using *B. thailandensis* as a model organism, Garcia et al. showed that delivery of BcpA-CT toxin into the immune (“self”) bacteria induces the gene expression and phenotypic changes within the recipient cells. This phenomenon was termed contact-dependent signaling (CDS) [27,56]. The changes due to CDI toxin delivery include, in particular, the upregulation of genes encoding pili and polysaccharide synthesis machinery, and promote biofilm formation. An effect on the autoaggregation ability and pigment production was also observed [13,56]. The molecular mechanisms underlying these changes are not yet understood; the reduced or residual BcpA-CT DNase activity, altered kinetics, or substrate specificity upon BcpI binding can be considered. Interactions of the toxin–immunity protein complex with target cell DNA or proteins could contribute to signaling (Figure 2, right) [13,28].

These facts could serve as a starting point for speculations about the possible communicative role of CDI systems: for example, one could imagine that each of the many variants of CDI toxins transmits certain information that can be “accepted” by neighboring cells only if an appropriate immunity protein is produced. The recipient cells not producing the cognate immunity protein will be destroyed. As in the case of quorum-based communication systems (QS), the signal intensity should be directly proportional to the size of the population, thus modulating the behavior of the whole community [13]. However, such arguments are still nothing more than speculation that requires experimental confirmation.

### 6.3. An Impact of CDI Toxins in Bacterial Populations

Although CDI systems have a well-established role in bacterial competition, there is increasing evidence that the function of these systems is not simply to antagonize related strains. Many findings allow suggesting that CDI systems may also facilitate and mediate interbacterial cooperation and collaboration. It has been shown that CdiA-like proteins (although some of them were not yet identified as CDI toxins in the earlier works) play roles in cell–cell aggregation or biofilm formation in several species including *Erwinia chysanthemii* [75], *Xylella fastidiosa* [76,77,78], *N. meningitidis* [79,80], *Xanthomonas axonopodis* [81], *B. thailandensis* [52,55], *E. coli* [82], and *P. aeruginosa* [61].

Biofilm formation is a prime example of cooperative behavior, in which individual bacteria collaborate to build multicellular communities. Such communities are beneficial because they protect single bacterial cells from predation and antimicrobial compounds. In *E. coli*, CdiA proteins facilitate the biofilm development by functioning as interbacterial adhesins, both through interactions with BamA and through receptor-independent interactions, possibly via CdiA–CdiA adhesion [82]. Disruption of the *cdi* locus in *B. thailandensis* abrogates biofilm formation [52]; additionally, *B. thailandensis* cells that constitutively express *cdi* genes produce more tight biofilm on glass coverslip than the mutant lacking the *bcpAIOB* locus [55].

Anderson et al. assumed that the CDI systems of *Burkholderia* are designed to competitively exclude “non-self” bacteria from pre-established biofilms through toxin delivery, and so alter the biofilm community composition of spatially structured populations [53]. Indeed, contacting with a cell of its own kind, the toxin is blocked by the corresponding immunity protein; if the cell is not related and there is no such protein, it is killed [83,84]. A mathematical model was proposed by Blanchard et al. which predicts that CDI-like competition systems may result in localized aggregation of inhibiting cells within one- and two-dimensional populations [84]. Using computational modeling and *E. coli* as an example, Bottery et al. showed that CDI systems have subtle and system-specific effects at the single-cell level, generating single-cell-wide boundaries between CDI-expressing inhibitor cells and their neighboring targets [24]. Together, all these studies demonstrate that specific toxin–immunity protein binding interactions can control a “self”/“non-self” discrimination in bacteria, and CDI mechanisms thus serve as a tool for recognizing genetically identical relatives in mixed bacterial populations. By recognizing related cells and destroying outsiders, CDI systems help maintain a species parity in a complex multispecies bacterial community like biofilms [53,55,56,82].

Some reports suggest that CDI systems are also important during bacterial exchanges that occur within host organisms. Though CdiA is not known to intoxicate eukaryotic cells, the CDI effectors can contribute to virulence at least due to their role in bacterial aggregation. Thus, CdiA homolog HecA of *E. chrysanthemii* promotes adherence to leaf epidermal cells and allows planktonic bacteria to join pre-established cell aggregates [75]. Similarly, CdiA homologs from *N. meningitidis* (HrpA) [80] and *X. fastidiosa* (HxfA) [78] are suggested to mediate initial adhesion to host cells. In [85], CDI toxins were shown to be involved in the generation of persister cells in bacterial populations that are presumably formed in the presence of CdiI through a feedforward cycle, in which the toxins induce the stringent response resulting in Lon-mediated degradation of the CDI immunity proteins and growth arrest. As we know, bacterial persistence is a transient physiological state in which persister cells are multidrug tolerant and thus able to effectively survive during antibiotic treatment, thus contributing to the pathogenesis of diseases.

## 7. Modulating the Microbial Communities: The Enemy of My Enemy Is My Friend

In recent decades, scientists have harvested an enormous amount of data from natural microbial communities, especially human-associated ones. This was facilitated by the explosive development of the molecular biological toolkit. Today, it is well known that oscillation within the human microbiome could have drastic consequences for the host health. Therefore, the research aimed to look for the ways to influence the structure and composition of microbial flora, which are of great importance now.

The microbial community is a stable ecosystem consisting of a huge diversity of microorganisms that can self-regulate. To maintain homeostasis, bacteria use a variety of mechanisms, both competition and cooperation, which were developed during a long evolution. CDI is one of the many tools that bacteria use to interact with each other in a complex microbial ecosystem, along with the well-known bacteriocins, as well as the relatively recently discovered “molecular syringe” (mediated by T6SS), or fratricide, which is a self-killing of a part of the bacterial population under stressful conditions. Thus, it is well known that the T6SS system is inherent in the *Bacteroidetes*, an abundant constituent of the gut microflora, which mediates interbacterial antagonism between cells nearby, at that the strain-specific competition with the involvement of T6SS of pathogenic enterotoxigenic *Bacteroides fragilis* with nontoxigenic *B. fragilis* was described [86,87,88]. Other examples are the observations that the probiotic strain *E. coli* Nissle 1917 uses antimicrobial peptides microcins to limit the expansion of competing *Enterobacteriaceae*, including pathogens such as adherent-invasive *E. coli* (AIEC) and *Salmonella enterica* ser. Typhimurium during intestinal inflammation [89], while oral commensal bacterial species *Streptococcus salivarius* produces a set of salivaricins, which can inhibit the growth of many oral pathogens including *Streptococcus pyogenes*, *Streptococcus pneumoniae*, and *Streptococcus mutans* [90]. An elegant example of the cooperative interaction between *S. pneumoniae* and *Haemophilus influenzae* which are the common commensals of the human airway and major bacterial pathogens of upper airway infections was done in [91] discovering the important role of such interaction in the pathogenesis of polymicrobial infections. It was shown that *H. influenzae* could promote pneumococcal survival, suppressing the expression of pneumococcal genes regulating autolysis and fratricide [91].

The last example shows that the mechanisms of interaction in microbial communities can be extremely complex. Unfortunately, in laboratory experiments, we still cannot reliably recreate the complex polymicrobial system and are limited to quite simplified models. Nevertheless, future studies are needed to further understand the mechanisms by which bacteria compete and cooperate, keeping in mind that such mechanisms may be shortly utilized as an alternative or complementary approach to the use of antibiotics. In the human body, the complex microbial ecosystem acts as a scaffold for dynamic cross-talk among and between beneficial and pathogenic microorganisms. Invasion or overgrowth of pathogenic species induces an instability (dysbiosis) that may directly influence the host physiology. For humans, it is beneficial when the commensal inhabitants restrain the reproduction of pathogenic species; however, when the microflora composition is unfavorable for humans, the idea to “push” some bacteria to maintain an appropriate balance is enticing. In other words, a philosophy of “wise monkey” who forces microbes within the community to fight with each other and watches it from atop a mountain can become the most profitable strategy in the near future.

## 8. Conclusions

Contact-dependent growth inhibition systems in bacteria are one more extremely interesting example of the microbial intra-community interaction in the context of “benefits-for-all” communities. To survive and overcome different biotic and abiotic pressures, bacteria must not only compete but also communicate and collaborate with their surrounding communities. Over the last decades, a lot of knowledge has been acquired regarding the strategies used by microorganisms for these purposes, including communication using quorum sensing or competition via diffusible bacteriocins production or close-contact “molecular syringes”. Discoveries continue to be made, prompting us to look for the impact of these phenomena on the whole microbial ecosystems. Several questions still require answers concerning the contact-depending bacterial interaction, such as whether the delivery of toxins is limited to cells of the same or close related species, and why this is the case, when the ability to inhibit the growth of others may provide a greater advantage. Novel experiments and model systems, including multispecies models, will be required to understand the contribution of CDI systems in shaping microbial communities. In conclusion, understanding how such systems work will contribute to an understanding of natural mixed bacterial communities and may provide new ways to manipulate the composition of growing communities.

## Figures and Tables

**Table 1:** CDI systems in bacterial species.

	Microorganism	CDI-Related Findings	Reference
*Escherichia*	*E. coli* strain EC93	The first description of the CDI phenomenon.	[32]
	*E. coli*	CDI toxins/receptors complexes.	[40,41,42]
	*E. coli*	Mechanisms of CDI regulation.	[28,35,36,38,43,47,48]
	*E. coli*, *B. pseudomallei*	CDI toxin/immunity protein complexes.	[30,37]
	*E. coli* strain 536 (UPEC536)	CysK enzyme stabilizes the complex of CdiA-CT with the immunity protein CdiI.	[44,49]
	*E. coli* strain NC101	CDI toxin/immunity protein/elongation factor Tu complex.	[50]
	*E. coli* strain Nissle 1917	Identification of functioning *cdiA-CT/cdiI* modules.	[51]
*Burkholderia*	*B. thailandensis*	The “*Burkholderia*” type of CDI locus is firstly defined. Expression of *bcpAIOB* genes is required for autoaggregation and adhesion on abiotic surfaces.	[52]
	*B. pseudomallei*	Ten subtypes of CDI systems within the *B. pseudomallei* species were identified.	[54]
	*B. thailandensis*	CdiA toxins probably participate not only in interbacterial competition but also in cooperation and recognition of “self” bacteria from “non-self”.	[53,55,56]
	*B. thailandensis*	The mechanism of CDI toxin delivery can differ even between closely related species.	[57]
	*B. dolosa*	Identification of three functioning CDI systems.	[58]
	*B. multivorans*, *B. thailandensis*	Non-pathogenic *B. thailandensis* uses CDI to control the growth of pathogenic *B. multivorans* during co-cultivation.	[59]
*Pseudomonas*	*P. aeruginosa* strain PAO1	Identification of multiple *cdi* loci in the *Pseudomonas* genomes. CDI systems are involved in the processes of adhesion and biofilm formation.	[61]
	*P. aeruginosa*	CDI system is vital for virulence of multidrug-resistant *P. aeruginosa* in acute/chronic infection.	[62]
	*P. aeruginosa*	Identification of CDI genes in *P. aeruginosa* genomes.	[63]
	*P. aeruginosa*	CDI systems have a toxic effect on mammalian cell culture and increase the virulence of *P. aeruginosa* strains in the mouse bacteremia model.	[64]
*Acinetobacter*	*A. baumannii*, *A. nosocomialis*	Identification of functioning CDI systems.	[65]
	*Acinetobacter* spp.	Identification of >40 variants of *cdi* loci within the genus *Acinetobacter*	[67]
	*A. baylyi* strain ADP1	Both variants of CDI systems discovered play no roles in biofilm formation or adhesion to epithelial cells.	[66]
	*A. baumannii* strain DSM30011	Identification of two types of CDI toxins. The functioning of CDI systems represses biofilm formation and adhesion to the host cells.	[68]
	*Neisseria meningitidis*	The crystal structure of the CdiI immunity protein.	[60]
	*Enterobacter cloacae*	CdiA-CT^ECL^/immunity protein complex.	[70]
	*Xenorhabdus doucetiae*	Identification of the *cdiBCAI* locus in the genomes of *Xenorhabdus* and *Photorhabdus luminescens*.	[71]
